# A study protocol of the effectiveness of the Attempted Suicide Short Intervention Program (ASSIP) for recent suicide attempters: a randomized controlled trial

**DOI:** 10.1186/s12888-024-06109-z

**Published:** 2024-10-04

**Authors:** Sara Lindström, Anna Ehnvall, Erik Bergqvist, Margda Waern, Marie Dahlin, Åsa Westrin

**Affiliations:** 1 Department of Clinical Sciences, Lund, Psychiatry, Lund University, SE-221 84 Lund, Sweden; 2 Office of Psychiatry and Habilitation, Region Skåne, SE-221 85 Lund, Sweden; 3 Department of Psychiatry and Neurochemistry, Institute of Neuroscience and Physiology, University of Gothenburg, SE-413 45 Gothenburg, Sweden; 4 Psychiatric Outpatient Clinic, Region Halland, SE-432 43 Varberg, Sweden; 5 Psychiatric In-patient Clinic, Hallands Sjukhus Varberg, Region Halland, SE-432 81 Varberg, Sweden; 6 Centre for Psychiatry Research, Department of Clinical Neuroscience, Karolinska Institute, & Stockholm Health Care Services, Region Stockholm, SE-112 81 Stockholm, Sweden; 7 Department of Psychiatry and Neurochemistry, Institute of Neuroscience and Physiology, University of Gothenburg, SE-413 45 Gothenburg, Sweden; 8 Department of Psychotic Disorders, Sahlgrenska University Hospital, Region Västra Götaland, SE-431 30 Mölndal, Sweden

**Keywords:** Suicide, Psychotherapy, Suicide prevention, Suicide attempters

## Abstract

**Background:**

Given the limited research focusing on psychotherapeutic interventions for suicide attempters, it is noteworthy that the Attempted Suicide Short Intervention Program (ASSIP) has demonstrated promising results in previous studies. In this investigation, we aim to evaluate the effectiveness of ASSIP across diverse healthcare settings, outlining the study design and planned evaluation.

**Methods:**

Using a Randomized Controlled Trial (RCT) design with four assessment points (baseline, 3, 12- and 24-month follow-up), we aim to assess the effect of the 3-session psychotherapeutic intervention and hereafter brief contact via structured letters during 2 years in a clinical sample of recent suicide attempters (suicide attempts within three months before inclusion). Participants are randomly assigned to one of two groups; treatment as usual plus ASSIP or the control condition, treatment as usual. Assessments include measures of suicidal intent, coping, symptoms of depression, quality of life, self-stigma, and sick leave. The primary outcome is suicide attempt(s) within 3, 12, and 24 months and the secondary outcome is suicidal ideation within the same time frames after study inclusion.

**Discussion:**

Findings from this study will provide novel insights regarding the effects of ASSIP on not only subsequent suicidal behavior but also other outcomes including self-stigma, quality of life, social network, sick leave, and symptoms of depression.

**Trial registration:**

The trial was registered at ClinicalTrial.gov NCT04746261 on 2020-10-15.

**Supplementary Information:**

The online version contains supplementary material available at 10.1186/s12888-024-06109-z.

## Background

In recent years, the global toll of suicide has exceeded 700,000 fatalities annually, with approximately 1,500 occurrences recorded in Sweden alone [1]. Nearly 11,000 individuals engaged in suicidal behaviors in Sweden in 2021, leading to hospitalization for at least one night or necessitating emergency services [2]. It is crucial to note that the actual prevalence of suicide attempts is likely underestimated, as not all instances come to the attention of healthcare professionals. Estimates suggest that only about half of the true number of suicide attempts receive hospital care in Sweden each year [3]. The gravity of suicide attempts as a risk factor for subsequent suicide is underscored by prior research [4]. Our research group has previously demonstrated that the risk of suicide persists over an extended period following an attempt, and is influenced by psychiatric disorders, suicidal intent, and number of attempts [5, 6]. Recognizing the history of suicide attempts as a high-risk demographic [7], it becomes imperative to prioritize comprehensive assessment, treatment, and ongoing monitoring after a suicide attempt.

In our investigations involving individuals who died by suicide in Sweden in 2015 and had prior contact with psychiatric services, it was revealed that 51% had a history of at least one suicide attempt [8]. Even though some of these individuals were receiving extensive pharmacological and psychological interventions, the persistence of suicidality underscores the inadequacy of existing treatments for this high-risk group. A study from the United States further illuminates this issue, reporting that 56% of individuals attempting suicide within 12 months had sought psychiatric services, yet half of them perceived unmet treatment needs [9].

Skogman et al. [10] conducted interviews with individuals who had previously attempted suicide. The authors underscored the importance of addressing patients’ desires for enhanced self-understanding, acquisition of problem-solving skills, improved help-seeking behaviors, and assistance with social and economic challenges. These identified needs align with resources such as psychotherapeutic interventions and multidisciplinary support systems. In a one-year follow-up, Cedereke et al. [11] investigated the needs of suicide attempt survivors. The authors concluded that post-attempt needs encompassed not only health-related aspects but also fundamental psychosocial needs, with social needs becoming more pronounced than psychiatric needs after a year. Synthesizing these insights, it becomes evident that suicide attempt survivors require a flexible, long-term follow-up strategy tailored to address a diverse array of needs.

Today, it is common practice in psychiatric services to document the grading of an individual’s suicide risk as an attempt to predict future suicide attempts or suicides, but such traditional suicide risk assessments lack clear scientific support [12]. Hawton and colleagues have proposed that instead of attempting to predict the risk of suicidal behavior, all patients with mental health problems should be considered potentially at increased risk of suicide, and tailored planning should be developed to manage each patient’s suicide risk [12]. This planning would involve not only assessing and summarizing the patient’s condition and suicide risks but also safety planning to prevent suicide attempts and other risk-reducing interventions such as contact with the appropriate healthcare provider, treatment, and psychosocial measures. There are various methods of safety planning [13] and research is ongoing regarding their evidence base and optimal utilization [14].

A recent comprehensive review encompassing 18 studies on psychotherapeutic interventions for suicide attempters found that Cognitive-Behavioral Therapy (CBT)-related and potentially psychodynamic approaches demonstrated efficacy in preventing subsequent suicide attempts [15]. This corroborates findings from a previous review [16] emphasizing that interventions should explicitly target suicidal behaviors/episodes rather than solely addressing symptoms of psychiatric disorders. Considering these findings, a pressing need for novel interventions emerges, tailored to the unique challenges faced by the group of high-risk individuals who have attempted suicide.

The Attempted Suicide Short Intervention Program (ASSIP), developed by suicide researchers Michel and Gysin-Maillart in Switzerland, emerges as a promising psychotherapeutic strategy for suicide prevention [17]. ASSIP includes a concise three-session format as an adjunct to standard care. Rooted in attachment theory, action theory, and cognitive theory, ASSIP recognizes the comprehensibility of patients’ actions through the lens of their life history, individual vulnerabilities, life goals, and basic needs. The intervention is designed to address the unique needs of each patient, supporting both short and long-term goals and structured as an add-on to regular treatment. The initial ASSIP session involves a narrative interview, videotaped for subsequent review. The second session includes video playback, enabling the patient and therapist to analyze and enhance the information jointly. Homework involves reading and commenting on a psychoeducational text covering themes that have been identified as relevant for understanding the background of suicide attempts in previous research. The third session involves the creation of a written case formulation, identifying individual vulnerabilities and the precipitating events leading to the suicidal crisis. Collaboratively, patient and therapist develop personalized suicide prevention measures. Long-term suicide prevention strategies, generated through individual analysis, may involve continued healthcare contact, including pharmacological and psychotherapeutic treatment. As part of the intervention, standardized letters from the therapist are sent to the patient over two years, to reinforce safety strategies, and offer support during potential crises.

In a randomized controlled trial (RCT) conducted in Switzerland [17], 60 patients receiving ASSIP in addition to standard care exhibited an 80% risk reduction for new suicide attempts within two years compared to the control group. Health economic benefits were observed [18]. However, a Finnish study [19] comparing ASSIP with crisis intervention demonstrated no discernible difference in effectiveness, which the authors potentially attributed to methodological limitations and the high prevalence of personality disorders among participants [19]. A small pilot RCT from the US [20] focusing on individuals with extensive substance abuse problems and suicide attempts, reported no difference in repeated suicide attempts but a reduction in suicidal thoughts with an adapted form of ASSIP treatment compared to standard care. Notably, the healthcare context appears to influence intervention outcomes. While ASSIP displays promising results, replication studies in diverse international contexts are hindered by methodological challenges. Comprehensive investigations, encompassing treatment fidelity, usual care content, and patient selection criteria, are warranted to determine the effectiveness of ASSIP in preventing new suicidal behaviors.

## Method

### Aims

In response to the scarcity of studies focusing specifically on psychotherapeutic interventions for individuals who have attempted suicide, our research endeavors to evaluate the efficacy of the Attempted Suicide Short Intervention Program (ASSIP) when implemented in psychiatric services for recent suicide attempters in Sweden. This paper outlines the study’s design and planned assessments, aiming to address key research questions.

Primary research questions include:

Efficacy of ASSIP: Does the incorporation of ASSIP into standard treatment effectively prevent new suicide attempts in individuals with a recent attempt compared to standard care only?

Identification of moderators and mediators: Can potential moderators or mediators associated with the efficacy of ASSIP be identified, contributing to a nuanced understanding of its impact?

Specific Patient Groups: Does ASSIP demonstrate efficacy in preventing new suicide attempts within specific patient groups, such as those diagnosed with depression, individuals with no attempt prior to the index attempt, or those who have made a serious suicide attempt?

Reduction of Self-Stigma: Does ASSIP contribute to a reduction in self-stigma among individuals who have attempted suicide?

Cost-effectiveness: Is the addition of ASSIP to conventional treatment cost-effective when compared to standard care?

Preliminary side projects include:

Self-Stigma Investigation: An exploration of self-stigma among suicide attempters, with a focus on assessing the potential impact of ASSIP on individuals’ experiences of stigma.

Coping Strategies Analysis: An investigation into coping strategies employed by suicide attempters, examining the correlation with stressful life events and exploring the potential influence of ASSIP on coping styles.

Adherence Scale Development: The development of an adherence scale to assess the extent to which ASSIP therapists adhere to the ASSIP manual, ensuring consistency and fidelity in the intervention’s implementation. Investigation of possible associations between adherence to the method and treatment outcome.

Mechanism of change within the ASSIP intervention: Investigation of whether the long-term therapy goals of the ASSIP conceptualization have been addressed and whether addressing these goals during follow-up impacts the primary and secondary outcome measures.

### Study design and data management

The study is an RCT. Patients are randomized to either receive ASSIP + usual treatment or only usual treatment. The project employs stratified randomization based on the study site, ensuring that 50% of patients receive ASSIP at all sites. Randomization occurs electronically at the end of visit 1 via REDCap and is blinded to the data handler. REDCap is an electronic data capture tool [21] hosted at Lund University which will be used to manage data collection. The participating regions will collectively recruit a total of up to 460 patients who have recently attempted suicide. For this study, a suicide attempt is operationally defined as a self-inflicted, potentially harmful behavior exhibiting explicit or implicit evidence of the individual’s intent to die [22].

### Clinical trial registration and registration date

The study is ongoing and was registered under ClinicalTrial.gov NCT04746261 on 2020-10-15 (https://clinicaltrials.gov/ct2/show/ NCT04746261), version 2, 2023-02-21. Further details are available on ClinicalTrials.gov under trial ID NCT04746261.

### Participants

Patients are recruited from diverse psychiatric healthcare settings, including psychiatric and emergency care, consultation psychiatry, inpatient psychiatric wards, and outpatient psychiatric care in Sweden. Those meeting the specified eligibility criteria (see below) are invited to participate in an initial visit. Ahead of the first visit, patients are provided with written information delineating the research project’s purpose, followed by a verbal description of its implications. All participant data will be kept in the REDcap system and in fire-safe locked cabinets at the research unit to ensure participant confidentiality. All data will be presented at group level so that no individual can be identified.

The study is currently recruiting.

### Eligibility criteria

Inclusion criteria

1. Age ≥ 18 years.
2. Recent contact with psychiatric healthcare following a suicide attempt within the three months preceding the first visit.
3. Scheduled meeting or appointment with a designated healthcare provider at a specific psychiatry or primary care unit after the initial visit to the study center.

Exclusion criteria

1. Current psychosis featuring active delusions, hallucinations, or negative symptoms likely to impact therapy.
2. Diagnosis of emotionally unstable personality disorder (ICD 10) as documented in the medical record.
3. Inability to undergo therapy without the assistance of an interpreter.
4. Intellectual disability, dementia, or any other condition impeding the comprehension of the study’s implications and hindering the provision of informed consent.

### Therapists

All therapists are experienced clinicians (psychologists, nurses, or social workers) at psychiatric services with a varying extent of prior training in psychotherapeutic techniques. All have experience of working with suicidal individuals. The ASSIP training includes two days of theoretical lectures and skills training as well as video examples. To become a certified ASSIP therapist, the trainee needs to complete five case treatments under supervision, all of which must be approved. The teachers and supervisors are the creators of the method or have been trained by the creators. Yet uncertified therapists who have full supervision may treat patients in the RCT.

### Interventions

The interventions being studied are ASSIP (described above in the background section) plus treatment as usual (TAU) or TAU only. TAU after a suicide attempt varies and is typically based on diagnosis, individual needs, and local healthcare routines. It may involve contact with primary care or specialized psychiatric services and various types of treatments such as pharmacological treatment, psychotherapy, or other interventions. For details of the interventions, see Table 1.

**Table 1:** Procedures for participants randomized to treatment as usual plus ASSIP

	Session 1	Session 2	Session 3	3 months after visit 3	6 months after visit 3	9 months after visit 3	12 months after visit 3	18 months after visit 3	24 months after visit 3
*Video recording of the session	X								
*Narrative interview	X								
*Video playback		X							
*Home assignment, either visit 1 or 2	X	X							
*Safety plan and Leporello**			X						
WAI-SR			X						
*Send standardized letters				X	X	X	X	X	X
Follow up according to the flow chart for all study participants				X			X		X

### Standardized evaluation forms for data collection

Throughout the project, a research evaluation is conducted during the initial baseline visit prior to randomization. These evaluations utilize:

- The Suicide Intention Scale (SIS) [23] is used to measure the severity of a specific suicide attempt. It assesses the intent behind the attempt, including factors such as planning, lethality, and perceived seriousness.
- Columbia-Suicide-Severity-Rating Scale (C-SSRS) [24] is a comprehensive tool used to assess past and recent suicidal ideation, behaviors, and actual suicide attempts. It evaluates the intensity and frequency of suicidal thoughts, as well as the presence of suicide plans and actual suicidal behavior. This scale provides a standardized method to assess suicide risk and monitor changes over time.
- Mini International Neuropsychiatric Interview (MINI)version 7.0.1 [25] is a structured diagnostic interview used for psychiatric assessments. It covers a wide range of mental disorders, including mood disorders, anxiety disorders, psychotic disorders, and substance use disorders. The MINI provides a standardized approach to diagnosis, allowing clinicians to identify psychiatric conditions based on established criteria.
- The Interview Schedule for Social Interaction (ISSI) [26] is a tool used to assess an individual’s social interaction patterns and skills through structured interviews. It evaluates various aspects of social interaction, such as communication, empathy, and assertiveness.
- AUDIT (Alcohol Use Disorders Identification Test) and DUDIT (Drug Use Disorders Identification Test) [27] are screening tools designed to identify individuals at risk of alcohol or drug-related problems. They consist of a series of questions assessing the frequency and intensity of alcohol or drug use, as well as related consequences.
- The Life Events Checklist for DSM-5 (LEC-5) [28] is a self-report measure used in assessing exposure to traumatic events according to the criteria of the Diagnostic and Statistical Manual of Mental Disorders (DSM-5). It helps clinicians or researchers gather information about various traumatic experiences a person may have encountered.
- The EuroQol 5-Dimension 5-Level (EQ-5D-5 L) [29] questionnaire is a standardized instrument for measuring health-related quality of life. It assesses five dimensions of health (mobility, self-care, usual activities, pain/discomfort, and anxiety/depression) using both descriptive items and a visual analog scale.
- The Montgomery-Åsberg Depression Rating Scale-Self Report (MADRS-S) [30] is a self-report measure used to assess the severity of depression symptoms. It consists of items that evaluate mood, vegetative symptoms, and cognitive symptoms commonly associated with depression.
- The Internalized Stigma of Mental Illness (ISMI) [31] self-report scale measures the degree to which individuals with mental illness internalize stigma. It assesses various aspects of self-stigmatization, including alienation, stereotype endorsement, perceived discrimination, social withdrawal, and stigma resistance.
- The COPE (Coping Orientation to Problems Experienced) self-report inventory [32] is a questionnaire designed to assess coping strategies used by individuals when dealing with stress or challenging situations. It measures various coping styles, such as problem-focused coping, emotion-focused coping, and avoidance coping.

The assessment is carried out by researchers associated with the project. The SIS, CSSR-S, and MINI assessments at the baseline visit are carried out by a specialist or resident in psychiatry. A trained research nurse with experience in clinical psychiatry conducts all other evaluations. These assessment tools provide a structured framework.

### Health encounters during the study

In addition, the study ensures that all participants have, see Table 2:

**Table 2:** Study procedures, all participants in the ASSIP RCT

	Baseline visit	3 months after the baseline visit	12 months after the baseline visit	24 months after the baseline visit	
Signing of the informed consent	X				
Demographic data*	X				
Background information*	X				
MINI 7.0.1	X				
C-SSRS	X	X	X	X	
SIS	X				
AUDIT self-report	X				
DUDIT** self-report	X				
LEC-5 self-report	X				
ISSI*** self-report	X	X	X	X	
EQ-5D-5 L*** self-report	X	X	X	X	
MADRS-S*** self-report	X	X	X	X	
ISMI*** self-report	X	X	X	X	ISMI*** self-report
COPE*** self-report	X				
Randomization to ASSIP + TAU or only TAU****	X				
Structured telephone interview according to the follow-up interview template		X	X	X	

*According to the interview template and self-report template

**For patients who answered yes in the interview regarding drugs

***Sent home by mail for follow-up at month 3, 12 och 24

****Patients who are randomized to ASSIP see “flow chart ASSIP therapy”

- scheduled follow-up healthcare engagement subsequent to the baseline visit.
- are contacted for follow-up sessions administered by a research nurse at 3, 12, and 24 months. These may include suicide prevention interventions as a precautionary measure to safeguard patient well-being.

While these are data points included in the research study (as described below baseline visits and follow-up visits), they also represent a minimal equivalent intervention that both groups receive, involving initial contact and basic follow-up.

### Study procedures

#### Baseline assessment

During the baseline visit, research interviews are administered by members of the research team, comprising a specialist or resident physician in psychiatry and an experienced mental health professional with clinical expertise in the targeted patient group. The research interview adheres to a structured interview protocol designed to systematically elicit information about various background factors, including but not limited to marital status, educational background, employment status, sick leave history, prior treatments, and healthcare contacts.

After the interview, the patient is randomized to either receive usual treatment plus ASSIP or only usual treatment, and the information about group assignment is provided to the patient in connection with the baseline visit, along with information about the appointment with the ASSIP therapist.

#### Outcomes

To assess the primary outcome variable of new suicide attempts, multiple data sources will be employed, and in the primary analyses, a comprehensive approach will be taken, combining data from patient reports via telephone interviews (including C-SSRS), medical charts, and registers. A participant will be considered to have a new attempt based on data from any source including lethal suicide attempts. The secondary outcome is suicidal ideation within the same time frames after study inclusion. Suicidal ideation is defined as MADRS-S item 9 ≥ 4 on self-assessment. All study participants undergo follow-up assessments at 3 months, 12 months, and 24 months after the initial visit through structured telephone interviews conducted by experienced healthcare professionals. During these follow-ups, details of treatments and interventions since the previous visit are recorded. In cases where ongoing suicidal ideation is detected, standard healthcare procedures are implemented to manage the situation within the established protocols. Follow-up assessors and outcome assessors are blinded. However, as the intervention is a psychotherapeutic treatment, it is obvious to participants and therapists that they are receiving the intervention and are therefore not blinded. No formal Data Monitoring Committee (DMC) will be involved in this trial as the REDCap system was considered adequate.The research team, including the principal investigators, will conduct periodic interim analyses to monitor the safety and effectiveness of the intervention. The research team will systematically monitor and document any adverse events throughout the trial. This trial does not include an external auditing process. The research team will ensure adherence to the protocol through internal monitoring and oversight. Regular meetings will be held to review study progress, data collection, and adherence to ethical and procedural standards.

To provide a comprehensive understanding of TAU, medical chart data are collected for the periods two years before and two years after the index suicide attempt.

Register data linked by personal identification number are compiled two years after the index/baseline from several sources, including:

1. The National Board of Health and Welfare’s Cause of Death Register: Data regarding suicide mortality.
2. Patient Register: Offering comprehensive information on inpatient and specialist healthcare utilization and diagnoses.
3. Prescription Drug Register: Detailing purchases of prescribed medications for the study participants.
4. Statistics Sweden (SCB): Longitudinal Integrated Database for Health Insurance and Labor Market Studies (LISA): Furnishing socio-economic and labor market-related data.

### Power calculation

Based on Swiss findings regarding the effect of ASSIP [17], we anticipate that 10% of individuals in the group receiving ASSIP plus conventional treatment, and 20% of individuals receiving conventional treatment alone, will attempt suicide again during the follow-up period. According to the chi-square test, 199 individuals are required in each group to achieve a significant difference with a power of 0.8 and an alpha value of 0.05. Assuming a 15% dropout rate among patients, 460 patients need to be recruited. If the dropout rate is lower, fewer patients will suffice.

### Mapping the implementation of ASSIP

Method adherence to the project is assessed by ensuring that each component of ASSIP has been implemented [19], and when implemented, it is recorded in a web-based application for electronic data collection in research studies. In collaboration with the creators of ASSIP, a scale has been developed to assess the adherence and competence of the therapists, ASSIP adherence and competence scale (ASSIP ACS) [33]. All therapists self-rate after each ASSIP session and overall method adherence will be rated by an independent rater who will review a random sample (10%) of all therapy sessions.

### Mapping of treatment as usual

TAU is mapped through telephone interviews and journal reviews using a specific template and recorded in REDCap. The mapping includes ICD-10 diagnoses, healthcare contacts, pharmacological and psychotherapeutic treatments, as well as other interventions.

### Statistical considerations

The chi-square test will be utilized to assess differences between the intervention group and the control group regarding the main outcome. The Wilcoxon signed-rank test is planned to be used to analyze significant changes over time, and the Mann-Whitney U test for differences between groups. To visually represent the cumulative probability of suicide reattempt over time in each treatment group, we plan to create Kaplan-Meier curves. Simple and multiple logistic regression to compare the relative impact of different factors on the outcome. Data regarding the professions and previous psychotherapeutic training of therapists will be collected and investigated as putative moderators. Further, we plan to employ cost calculation items for assessing direct and indirect costs, and total costs for the cost-effectiveness model.

### Reporting and publication plan

In accordance with the Declaration of Helsinki, study results will be made publicly available, through publication and/or a public database as soon as possible after the study is completed. This applies regardless of whether the results are positive, negative, or neutral. The first main outcome study will provide preliminary results after a one-year follow-up is completed for the last participant included in the RCT.

## Discussion

This large-scale Swedish intervention study, conducted as an RCT, aims to investigate the effects of a brief psychotherapeutic intervention known as ASSIP within psychiatric services for individuals who have recently attempted suicide. The study design is poised to offer novel insights not only into the direct impact of ASSIP on reducing suicide attempts but also into a wide array of potential mediators and moderators. These include various aspects of mental health, therapeutic alliance, coping strategies, social support networks, experiences of stigma, sick leave patterns, and the specific components of the ASSIP intervention.

Should this suicide prevention intervention trial yield positive outcomes, it stands to build upon prior research findings related to the prevention and treatment of individuals who have recently attempted suicide, potentially elucidating the underlying mechanisms of action of ASSIP.

Through this comprehensive approach, our study endeavours to provide valuable insights into the efficacy factors that influence outcomes, the cost-effectiveness of ASSIP, and its potential benefits across diverse patient populations. Ultimately, our research aims to advance the understanding of psychotherapeutic interventions for suicide prevention.

## Electronic supplementary material

Below is the link to the electronic supplementary material.

Supplementary Material 1

## Data Availability

No datasets were generated or analysed during the current study.

## Abbreviations

ASSIP: Attempted Suicide Short Intervention Program
ASSIP-ACS: Attempted Suicide Short Intervention Program - Adherence and Competence Scale
AUDIT: Alcohol Use Disorders Identification Test
C-SSRS: Columbia-Suicide Severity Rating Scale
COPE: Coping Orientation to Problems Experienced
DSM-5: Diagnostic and Statistical Manual of Mental Disorders, 5th Edition
DUDIT: Drug Use Disorders Identification Test
EQ-5D-5L: EuroQol 5-Dimension 5-Level
ICD-10: International Classification of Diseases, 10th Revision
ISMI: Internalized Stigma of Mental Illness
ISSI: Interview Schedule for Social Interaction
LEC-5: Life Events Checklist for DSM-5
LISA: Longitudinal Integrated Database for Health Insurance and Labour Market Studies
MADRS-S: Montgomery-Åsberg Depression Rating Scale - Self Report
MINI: Mini International Neuropsychiatric Interview
RCT: Randomized Controlled Trial
SCB: Statistics Sweden (Statistiska centralbyrån)
SIS: Suicide Intention Scale
TAU: Treatment As Usual
WAI-SR: Working Alliance Inventory - Short Revised
